# Preparation of uranium(III) in a molten chloride salt: a redox mechanistic study

**DOI:** 10.1007/s10967-018-5953-7

**Published:** 2018-06-28

**Authors:** Hugues Lambert, Timothy Kerry, Clint A. Sharrad

**Affiliations:** 1 School of Chemical Engineering and Analytical Science, The University of Manchester, Oxford Road, Manchester, M13 9PL UK; 2 Present Address: Lhoist Recherche et Développement, Business Innovation Cente, 31, rue de l’Industrie, 1400 Nivelles, Belgium; 3 Present Address: Department of Materials Science and Engineering, Delft University of Technology, Mekelweg 2, 2628 CD Delft, The Netherlands

**Keywords:** Uranium(III), Molten salts, Electronic absorption spectroscopy, Cyclic voltammetry, Open circuit potential

## Abstract

The most advanced methodology for the pyroprocessing of spent nuclear fuel is the electrorefining of uranium metal in LiCl–KCl eutectic, in which uranium is solubilized as U(III). The production of U(III) in LiCl–KCl eutectic by the chlorination of uranium metal using BiCl_3_ has been performed for research purposes. In this work, this reaction was studied in-situ by visual observation, electronic absorption spectroscopy and electrochemistry at 450 °C. The most likely mechanism has been determined to involve the initial direct oxidation of uranium metal by Bi(III) to U(IV). The dissolved U(IV) then reacts with unreacted uranium metal to form U(III).

## Introduction

**Fig. 1:**
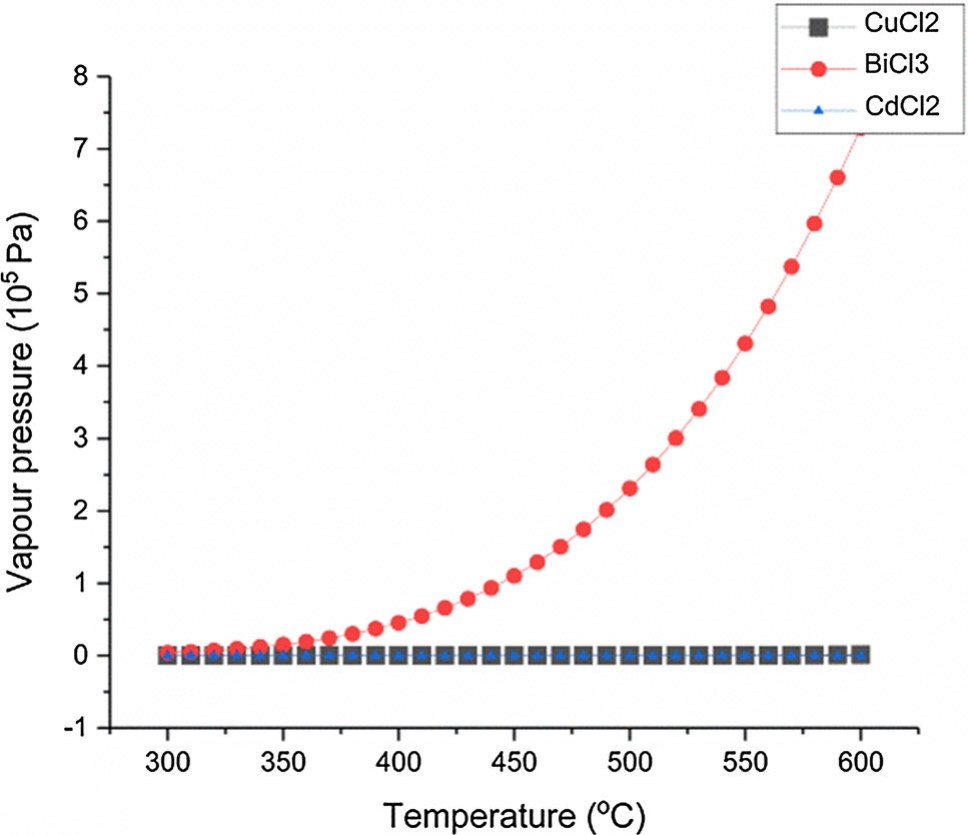
Plot of vapor pressure vs temperature for different chlorinating agents; black: CuCl_2_; red: BiCl_3_; blue: CdCl_2_. Calculated from the HSC Thermochemical Database. (Color figure online)

With a renewed interest in pyrochemical technology for spent nuclear fuel treatment [1–3] and molten salt reactor reprocessing schemes [4, 5], fundamental studies in molten salt chemistry are becoming key to understanding engineering scale experimentation and to develop process modelling [6, 7]. Pyroprocessing is a possible alternative to the more traditional hydrometallurgical route for spent nuclear fuel treatment, especially for some GEN IV fuels such as nitrides and metallic fuels. Its main advantages are the radiation resistance of molten salt and minimum criticality risk enabling a more compact process. In the 1980s Argonne National Laboratory developed a process at pilot scale to treat spent metallic fuel from its EBR-II reactor as part of the Integral Fast Reactor project [8]. The core of the reprocessing scheme is the electrotransport of uranium via anodic dissolution in a molten LiCl–KCl–UCl_3_ (5 wt% U) salt at 500 °C. During this operation the dissolved uranium is in contact with uranium metal. The presence of uranium metal will control the redox potential of the salt and the dissolved uranium will be present as U(III). While most chemical elements can be purchased as dry chlorides, it is not the case for uranium. In order to study this system it is important to established a reproducible route to prepare LiCl–KCl eutectic (LKE) containing U(III). The most common forms of uranium are as nitrates, oxides and metal, all in oxidation states other than +III. Uranium metal can be used for the production of dry chloride in different ways, but most of those methods would use corrosive gases and/or produce harmful volatiles [9–11]. It is important to underline the sensitivity of uranium chlorides towards oxygen and water [12]. Therefore, classical synthetic production routes producing powders with high surface area [13] are ill-suited for facilities where it is difficult to ensure an inert atmosphere at all times. The oxidation of uranium metal by metal chlorides is a preferred route for the production of chloride salt containing U(III). It also enables the direct dissolution of uranium chloride in the salt. Once quenched, the dissolved U(III) chloride is relatively well-protected from oxidation by air/moisture from the atmosphere. PbCl_2_ was proposed for the synthesis of U(III) in LKE as early as 1959 [14]. But historically, CdCl_2_ has been used in the Mark IV electrorefiner at the Idaho National Laboratory (INL) for direct production of uranium trichloride in molten LKE [15, 16]. Its two main drawbacks are the presence of cadmium, which raises toxicity concerns, and difficulties in removing any remaining CdCl_2_ at the end of the reaction by volatilization. To overcome those difficulties, INL has been studying alternative chlorinating agents such as CuCl_2_ [17]. Unfortunately the formation of UCl_4_ as an intermediary product makes this reaction incompatible for industrial processes as UCl_4_ reacts with iron to form FeCl_2_. Furthermore, the low vapour pressure of CuCl_2_ at the typical temperatures required makes it difficult to apply in the laboratory. In the early 2000s, the use of BiCl_3_ was proposed for the oxidation of plutonium metal in LKE at laboratory scales [18]. This route is efficient, convenient and the only two contaminants are bismuth metal, which will be inert at the bottom of the reaction vessel and non-reacted BiCl_3_. Another reason for the use of BiCl_3_ in laboratory preparations of actinide(III) chlorides is the fact that its vapour pressure is higher than that for CdCl_2_, as shown in Fig. 1, and is therefore much easier to volatize at the end of the reaction. This synthetic approach has also been successfully applied to the production of UCl_3_ and NpCl_3_ [19, 20].

The calculated Gibb’s energy of reactions likely to occur from reacting U metal with BiCl_3_ are presented below (Eqs. 1–4) [21]. The direct formation of UCl_4_ appears to be favoured but these calculations are limited as the exact molecular species in the solution melts are not considered.

1 $$ {\text{U}}_{\text{metal}} + {\text{BiCl}}_{3} \to {\text{UCl}}_{3} + {\text{Bi}}_{\text{metal}} \quad \mathop \Delta \limits_{r} G\left( {450\;{^\circ }{\text{C}}} \right) = - 471{\text{ kJ mol}}^{ - 1} $$

2 $$ {\text{U}}_{\text{metal}} + \frac{4}{3}{\text{BiCl}}_{3} \to {\text{UCl}}_{4} + \frac{4}{3}{\text{Bi}}_{\text{metal}} \quad \mathop \Delta \limits_{r} G\left( {450\;{^\circ }{\text{C}}} \right) = - 492{\text{ kJ mol}}^{ - 1} $$

3 $$ {\text{UCl}}_{3} + \frac{1}{3}{\text{BiCl}}_{3} \to {\text{UCl}}_{4} + \frac{1}{3}{\text{Bi}}_{\text{metal}} \quad \mathop \Delta \limits_{r} G\left( {450\;{^\circ }{\text{C}}} \right) = - 21{\text{ kJ mol}}^{ - 1} $$

4 $$ {\text{U}}_{\text{metal}} + 3{\text{UCl}}_{4} \to 4{\text{UCl}}_{3} \quad \mathop \Delta \limits_{r} G\left( {450\;{^\circ }{\text{C}}} \right) = - 409{\text{ kJ mol}}^{ - 1} $$

A similar method has been used to produce U(III) in fluoride melts [22]. This work presented here extensively explores the reaction of U metal with BiCl_3_ in LiCl–KCl eutectic at 450 °C using electrochemical and spectroscopic techniques to probe the redox mechanistic behavior of this reaction, building upon previous work by Bae et al. [23], in order to ensure the control and efficiency of this key reaction for the production of U(III) as the research of actinide behavior in molten salt systems expands with advancing nuclear technologies.

## Experimental

### Apparatus and electrochemistry

The furnace and the spectroscopic cell used in these studies have been described elsewhere [8]. A three electrode system was used for all electrochemical studies, unless otherwise stated, composed of two tungsten rods (2 mm diameter) as working and counter electrodes and a reference electrode of Ag/AgCl housed in mullite. In order to fit the electrodes in the cell, an in-house PTFE holder was designed with four insertion holes. The holes were used to house the three electrode system and an additional tube used, if necessary, to insert material, bubble gases and/or extract the melt for quenching. All electrochemical experiments were controlled using a PGSTAT 101 potentiostat from Metrohm. All potentials are reported vs Ag/AgCl (1.5 wt% in LKE).

### Electronic absorption spectroscopy

**Fig. 2:**
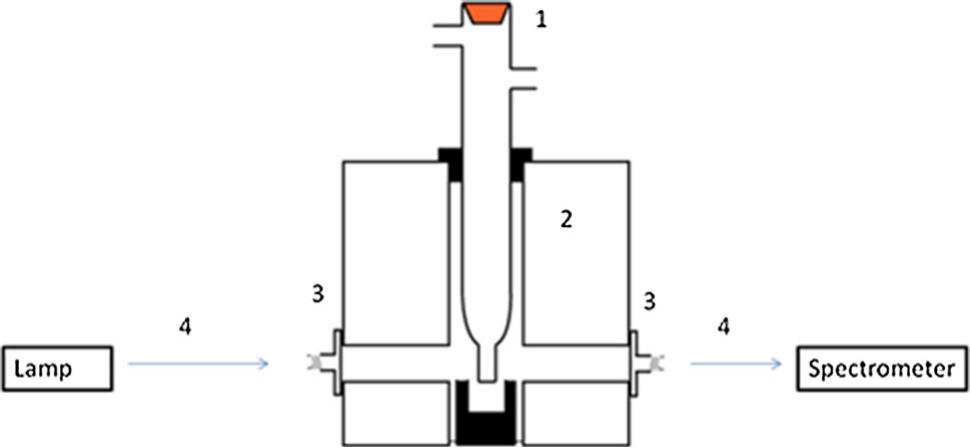
Schematic diagram of the spectroscopic set-up; 1: cell; 2: furnace; 3: collimator; 4: fiber optic

In-situ high temperature electronic absorption spectroscopy of the melts was performed using the set-up displayed in the schematic diagram shown in Fig. 2. The light from a tungsten halogen lamp source is directed to the furnace via fiber-optics. Two types of optical fiber were used depending on the application:

- Solarized UV–vis fiber optics from AVANTES for use from 180 to 900 nm.
- Broadband vis–NIR fibre optics from AVANTES for use from 500 to 1600 nm.

The fiber is connected to a collimator that ensures the beam of light is directed through the cell. The light is collected back from a similar collimator into the fibre optic which is connected to the spectrometer. Depending on experimental requirements, three spectrometers were utilised:

- UV detector (S1), AVASPEC-UL2048L, working from 180 to 640 nm.
- Vis detector (S2), AVASPEC-UL2048XL-RS, working from 500 to 1100 nm.
- NIR detector (S3), AVASPEC-NiR256 1.7, working from 950 to 1600 nm.

This enabled the study of the electromagnetic spectrum from 180 to 1600 nm.

### Salt preparation

LiCl (Alfa Aesar, 99% min) and KCl (Sigma, 99% min) were mixed in 45–55 wt% ratios and added to an alumina crucible. The powder was dried, at 140 °C, under vacuum overnight in a tube furnace. The temperature of the tube furnace containing the salt mixture was raised to 450 °C and held for three hours under argon. The purity of the salt was determined by cyclic voltammetry, by assessing the oxygen and/or hydroxyl anion content in the salt using a tungsten electrode. If necessary, the salt was electrolyzed [24] using a vitreous carbon rod as a working electrode, a tungsten rod as the counter electrode and Ag/AgCl (1.5 wt% in LKE) sheeted in mullite as a reference electrode, to remove any transition metal impurities and remaining water/oxygen content in the salt. The molten salt was then quenched in a quartz tube and kept under an argon atmosphere before use.

### Reagent preparation

BiCl_3_ (anhydrous beads 99.999% Sigma) was used as received. The uranium metal was obtained from the Centre for Radiochemistry Research (University of Manchester) stocks. The uranium metal was contacted with concentrated nitric acid (~ 10 M) in accordance with previously described procedures to remove the corroded surface layer [25]. The cleaned metal was immediately rinsed in distilled water followed by acetone. The metal sample was patted dry upon paper towel and immediately added to the cell, where any remaining moisture/acetone was removed under vacuum.

### Synthesis of U(III) in LiCl–KCl eutectic

At room temperature, the dried salt (16.70 g) and the uranium metal (0.30 g) were further dried under vacuum in the quartz cell at 140 °C for two hours, using the tube furnace. The electrodes were inserted into the cell, but held above the salt mixture, and the furnace temperature was then raised until the salt temperature was 450 °C. Once the salt was molten, the electrodes were lowered in the liquid salt and the absorption spectrum of the melted salt was acquired. BiCl_3_ (0.30 g) was directly added to the liquid melt, using the insertion tube. The reaction was left to evolve for eight hours after which, argon was bubbled in the solution in order to bring the reaction to completion. The open circuit potential (OCP) of the salt was recorded from the point the BiCl_3_ addition was made. Cyclic voltammetry was also performed at regular time intervals in order to follow the salt composition. The reaction was monitored using absorption spectroscopy and observations were made from visual inspection of the molten salt mixture in the quartz cell. This experiment was performed multiple times and negligible differences were observed for the overall trends in results obtained using both spectroscopic and electrochemical techniques. However, the observed kinetics of this reaction did vary between experimental runs.

## Results and discussion

### Visual observations

**Fig. 3:**
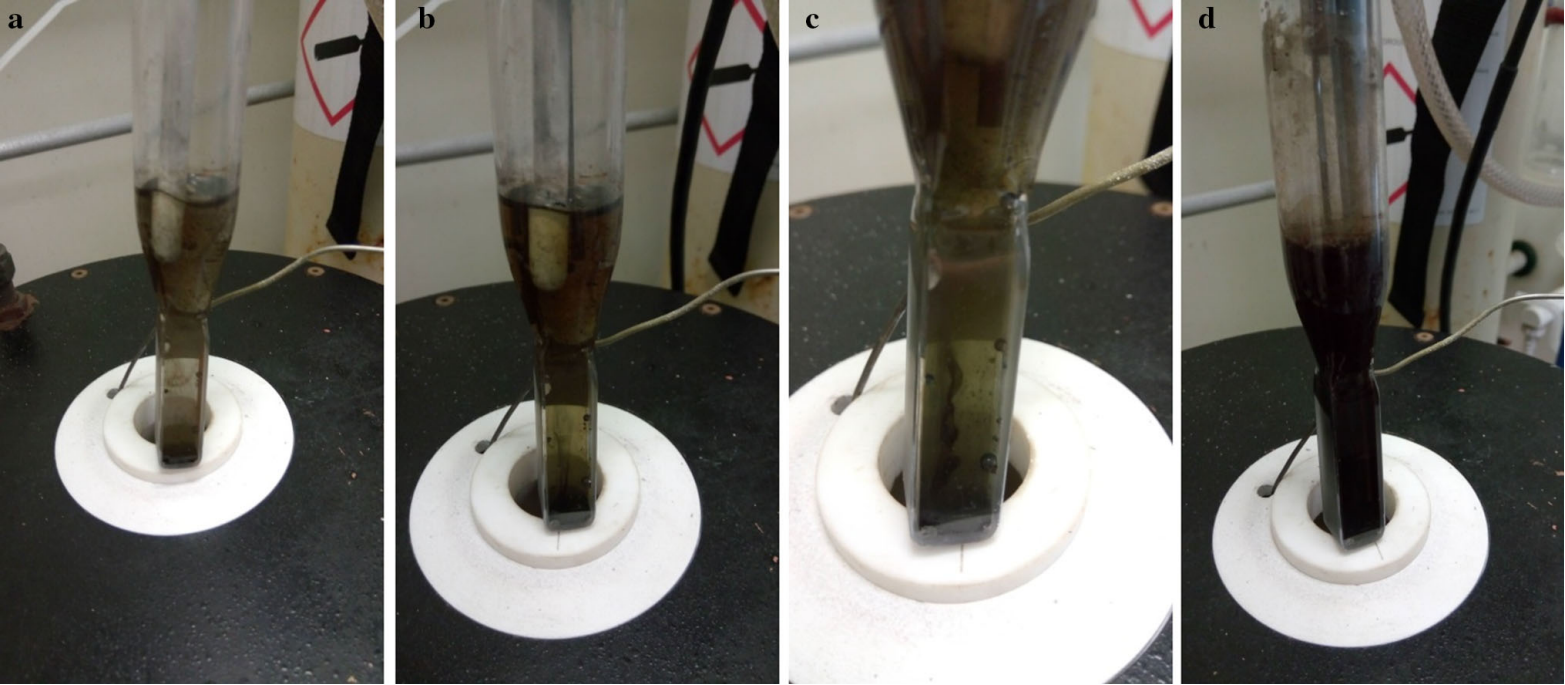
Pictures showing an example of the reaction progression of U metal with BiCl_3_ in LiCl–KCl eutectic at 450 °C over time; **a** 135 min; **b** 380 min; **c** 470 min; **d** 500 min

Contact of the salt with U metal without any other reagents showed the salt remained transparent indicating that no reaction had taken place. As BiCl_3_ was added, no immediate change was noted. Typically after approximately one hour, the salt was a green colour, as shown in Fig. 3a, b, indicating the presence of U(IV) in solution [9]. The increasing presence of a metal pool at the bottom of the cell was observed as the reaction continued. As the green coloration of the salt became more intense, the presence of a “smog chimney” through the melt was noted. The smog appeared to be emanating from the still reacting uranium metal sample located at the bottom of the cell. This dark “smog” is most likely due to the formation of U(III) which is known to be a very intense purple colour in chloride melts [9]. As the reaction proceeded, the intensity of this “smog” increased as shown in Fig. 3b, c, but still was observed in a “chimney” form suggesting that as the U(III) diffused into the bulk of the melt it was being converted to U(IV). Typically after approximately four hours of the reaction, the metal pool at the bottom of the cell was several millimetres thick. The exact timings of these observations did vary between experimental runs which we attribute to a number of factors such as different surface area: volume ratios of the uranium metal used in the reaction, differences in the quality of the “cleaned” uranium metal surface and avoiding a mixed reaction system. However, the order and nature of observations made during the progression of this reaction were consistent across experiments. Subsequent thorough mixing of the reaction solution by the bubbling of argon resulted in the salt becoming the deep-purple colour typical for U(III) as shown in Fig. 3d [9].

### Electronic absorption spectroscopy

**Fig. 4:**
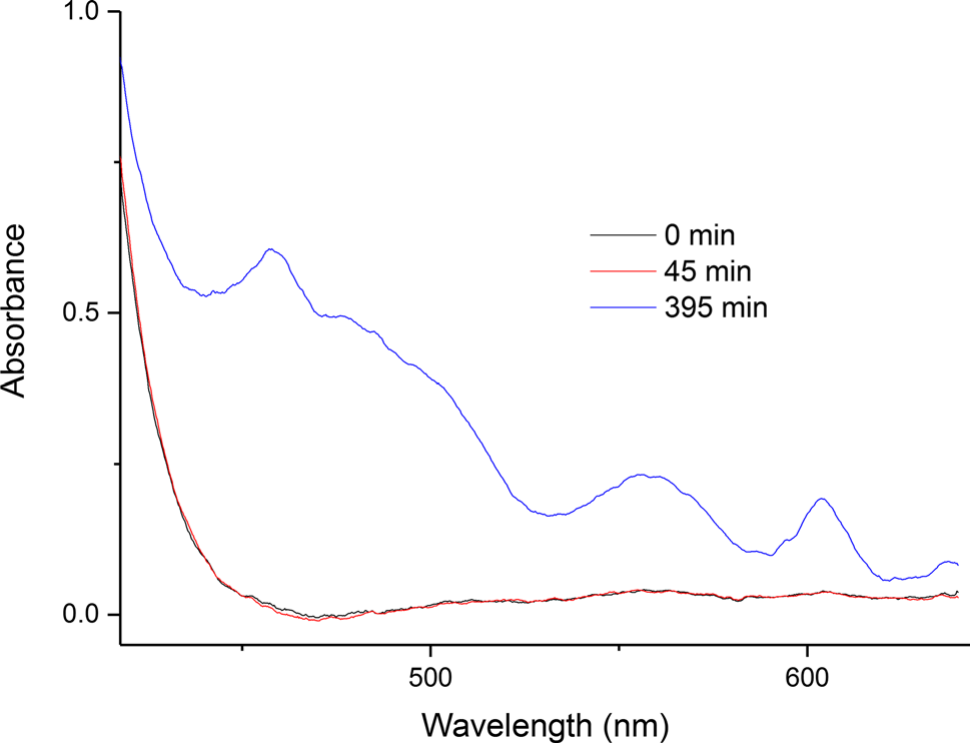
Electronic absorption spectra of a reaction of U metal with BiCl_3_ in LiCl–KCl eutectic salt at 450 °C. Evolution of the spectra in the 400–640 nm region was measured using detector S1. Times are indicative of the reaction progression for this specific experimental run

**Fig. 5:**
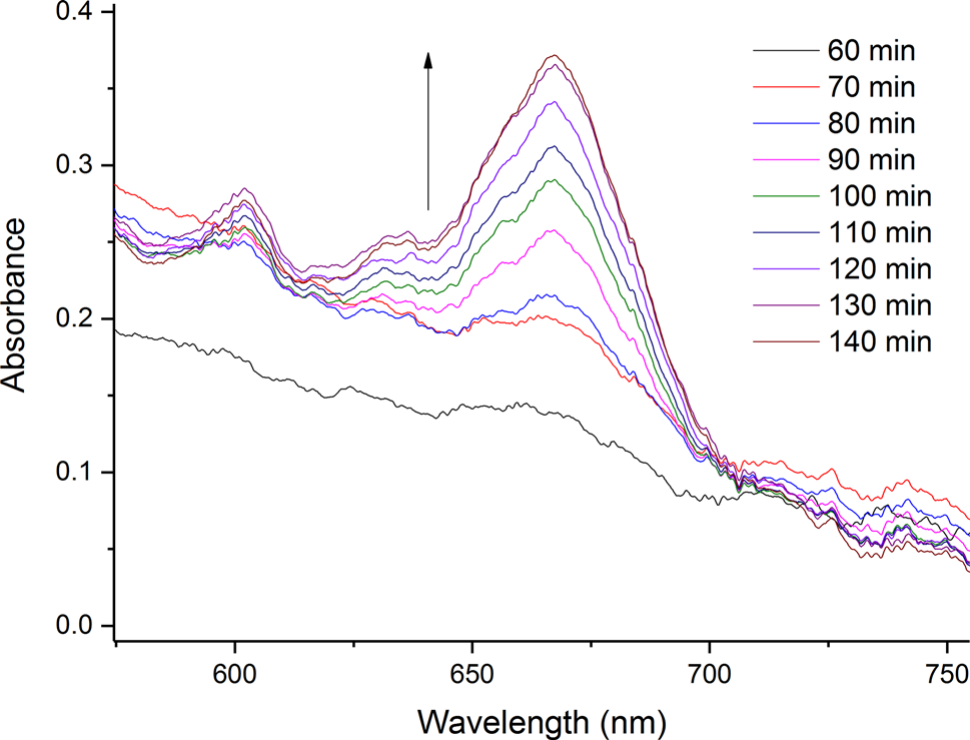
Electronic absorption spectra of a reaction of U metal with BiCl_3_ in LiCl–KCl eutectic salt at 450 °C. Evolution of the spectra in the 550–800 nm region was measured using detector S2. Times are indicative of the reaction progression for this specific experimental run

In-situ electronic absorption spectra of the molten salt were acquired employing a spectroscopic system similar to those used previously to acquire absorption spectra of high temperature melts [9, 26, 27]. The incoming light beam was positioned ~ 1 cm above the uranium metal sample in order to attempt to identify any semi long-lived intermediate species in the melt during the reaction, but sufficiently far away from the metal sample to avoid light scattering from the Bi metal that formed. Spectra were obtained at various time intervals as the reaction of U metal with Bi(III) proceeded. The relatively close proximity of the beam position to the uranium metal sample was chosen in order to possibly identify the formation of any intermediary products from this reaction. Immediately after the addition of BiCl_3_, a peak appears in the UV region and a saturated band can be observed up to 420 nm, which is concordant with the dissolution of Bi^3+^ in chloride media [28, 29]. During the initial stages of the reaction, spectra were recorded from 180 to 640 nm, using detector S1, in order probe for the intensely absorbing U(III) f → d transitions at 458 and 555 nm [30]. As shown in Fig. 4, none of the characteristic U(III) peaks appeared during the first 60 min after BiCl_3_ was added to the melt for this experiment. However, peaks were observed at 460, 561 and 604 nm which are characteristic of U(IV) in chloride melts [30], and have been observed in previously studies reactions of U metal with BiCl_3_ in LiCl–KCl eutectic melts. After approximately one hour, the detector S2 was connected in order to follow the evolution of the spectra in the visible-nIR region from 500 to 1025 nm for the remainder of the reaction. This enabled the following of U(IV) formation through its most intense absorption peak at 670 nm [8]. This is shown in Fig. 5. Despite the visual observation of a “smog chimney” in the melt emanating from the reacting U metal sample most likely due to the formation of U(III), no evidence of U(III) formation was observed in the spectra acquired while the melt was not mixed.

**Fig. 6:**
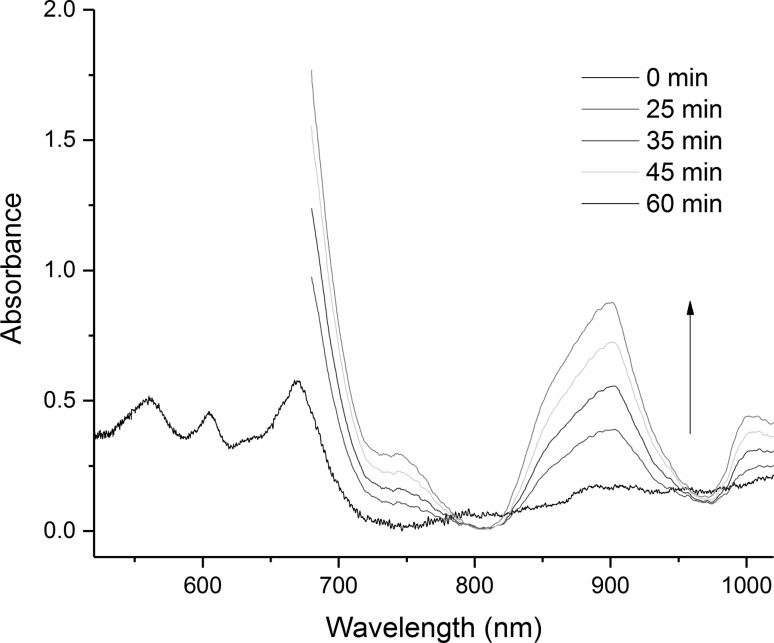
Electronic absorption spectra of the reaction of U metal in contact with U(IV) during the overall reaction of U metal with BiCl_3_ in LiCl–KCl eutectic at 450 °C. Times are indicative of the reaction progression for this specific experimental run

Figure 6 shows the evolution of the spectra from a reaction of the remaining U metal with the dissolved UCl_4_ in LiCl–KCl eutectic at 450 °C. The spectra evolves from a characteristic U(IV) profile towards that of U(III). The U(III) peak at 899 nm increases in intensity.

**Fig. 7:**
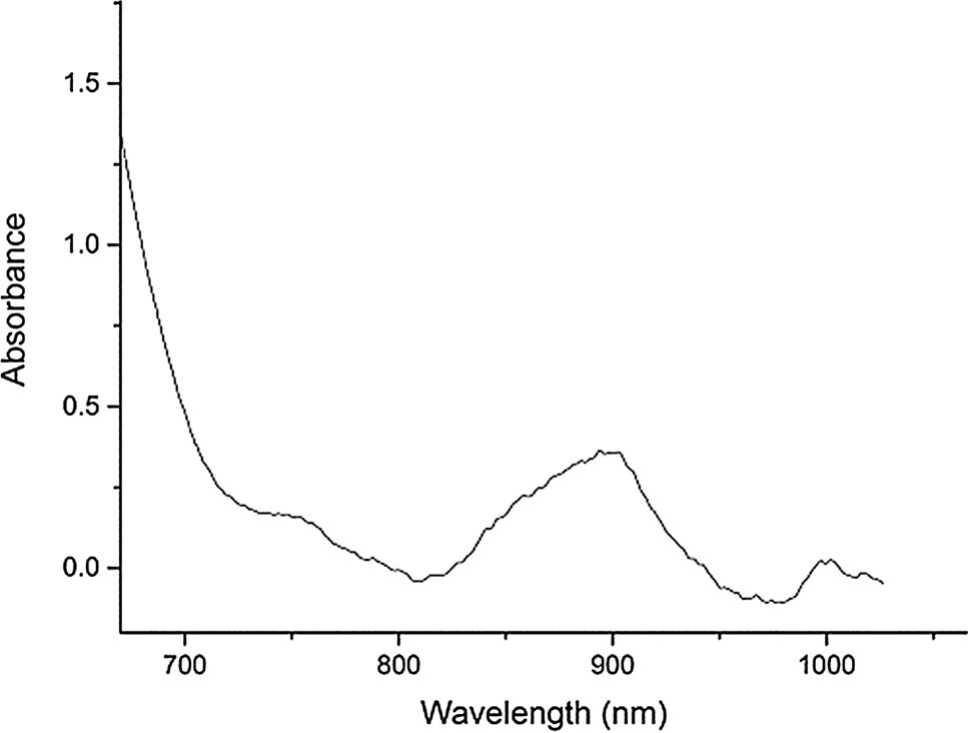
Electronic absorption spectrum of the reaction mixture of U metal with BiCl_3_ in LiCl–KCl eutectic at 450 °C after agitating the melt with Ar gas bubbling. The spectrum was measured with a quartz rod inserted in the cell using the spectrometer S2

After the bubbling of argon, the spectroscopic profile became saturated across the entire UV–visible region of the spectrum as is expected for high U(III) concentrations [27]. Consequently, a quartz rod, 6 mm diameter, was inserted into the cell in order to reduce the solution pathlength. This produced the spectrum shown in Fig. 7. Even with a quartz rod in the cell, the region below 700 nm is saturated but a peak is identified at 899 nm which is characteristic of U(III) [27, 31, 32].

### Electrochemistry

**Fig. 8:**
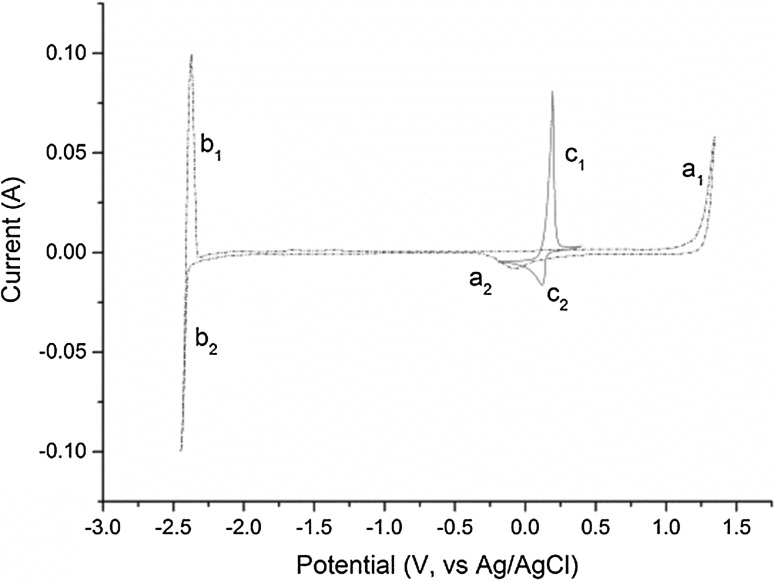
Cyclic voltammograms of clean LKE salt (dashed black line; Assigned peaks—a1: Cl^−^ → ½Cl_2_ + e^−^; a2: ½Cl_2_ + e^−^→Cl^−^; b1: Li → Li^+^ + e^−^; b2: Li^+^ + e^−^→Li) and BiCl_3_ (0.30 g) added to LKE (16.70 g) one hour after addition (red line; Assigned peaks—c_1_: ${\text{Bi }} \to {\text{Bi}}^{{ 3 { + }}} + {\text{  3e}}^{ - }$; c_2_: ${\text{Bi}}^{{ 3 { + }}} + {\text{  3e}}^{ - } \to {\text{Bi}}$) {T = 450 °C; Scan rate = 0.1 V s^−1^; Working electrode: W rod (S = 3 × 10^−4^ cm^2^); Counter electrode: W rod; Reference electrode: Ag/AgCl (1.5 wt% in LKE)}. (Color figure online)

**Fig. 9:**
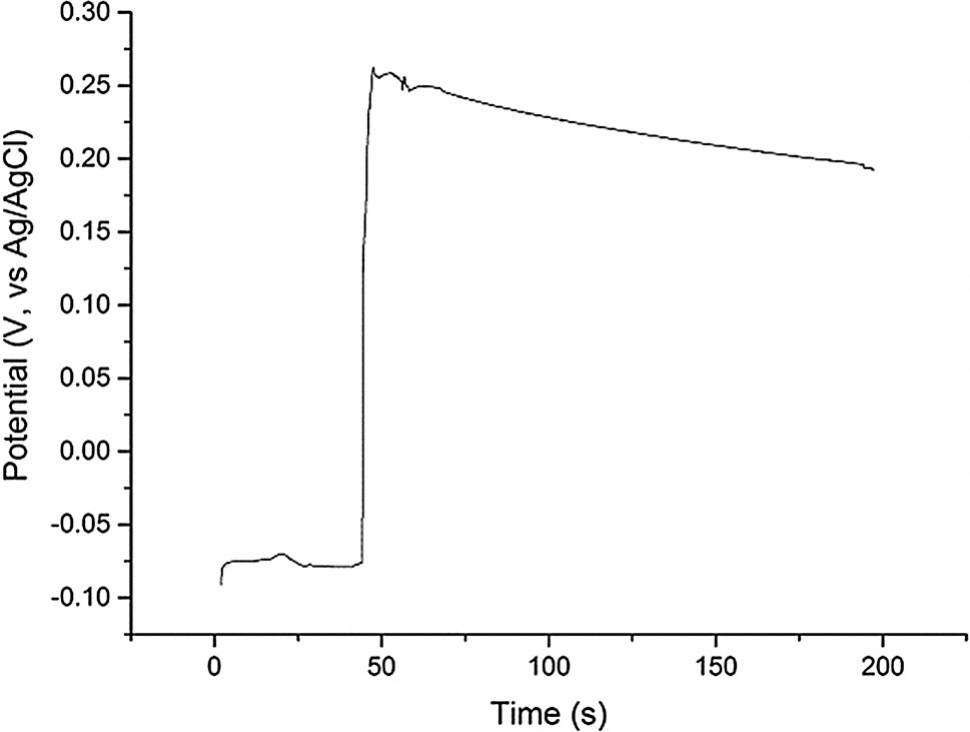
Plot of Open Circuit Potential (vs Ag/AgCl) from LiCl–KCl eutectic at 450 °C with U metal after BiCl_3_ addition (at t ~ 45 s) with respect to time

Before adding BiCl_3_, the purity of the salt was confirmed by cyclic voltammetry (CV) with no peak observed within the electrochemical window of the salt (Fig. 8). Figure 9 shows the increase in the Open Circuit Potential (OCP) after the BiCl_3_ addition, with time zero corresponding to the start of the measurement and the BiCl_3_ was added on the top of cell 30–60 s after the OCP started to be recorded. The red curve in Fig. 8 is a cyclic voltammogram recorded one hour after the addition of BiCl_3_ which shows the presence of an additional peak at 0.15 V (vs Ag/AgCl), which is concordant with the Bi/Bi^3+^ couple in LiCl–KCl eutectic [23, 33]. The boiling point of BiCl_3_ (447 °C) is lower than the temperature employed in these studies so it was important to verify the stability of Bi(III) in the melt. Indeed in our studies we have observed that BiCl_3_ remains solubilised in molten LiCl–KCl eutectic over long time periods (~ 10 h) for temperatures up to 500 °C. Volkov et al. [28] have shown by absorption spectroscopy that BiCl_3_ is dissolved as BiCl_4_^−^ in LiCl–KCl eutectic explaining its stability. After the sudden increase of the OCP in the melt, with the addition of BiCl_3_, the OCP then steadily decreases reflecting a decrease in the Bi(III) concentration in the melt either as the reaction with U metal proceeded, or through loss by volatilization. The latter possibility is unlikely as no condensation of any solid material was observed anywhere in the cell during the reaction, especially parts of the cell protruding from the furnace which would be at temperatures substantially lower than 447 °C.

**Fig. 10:**
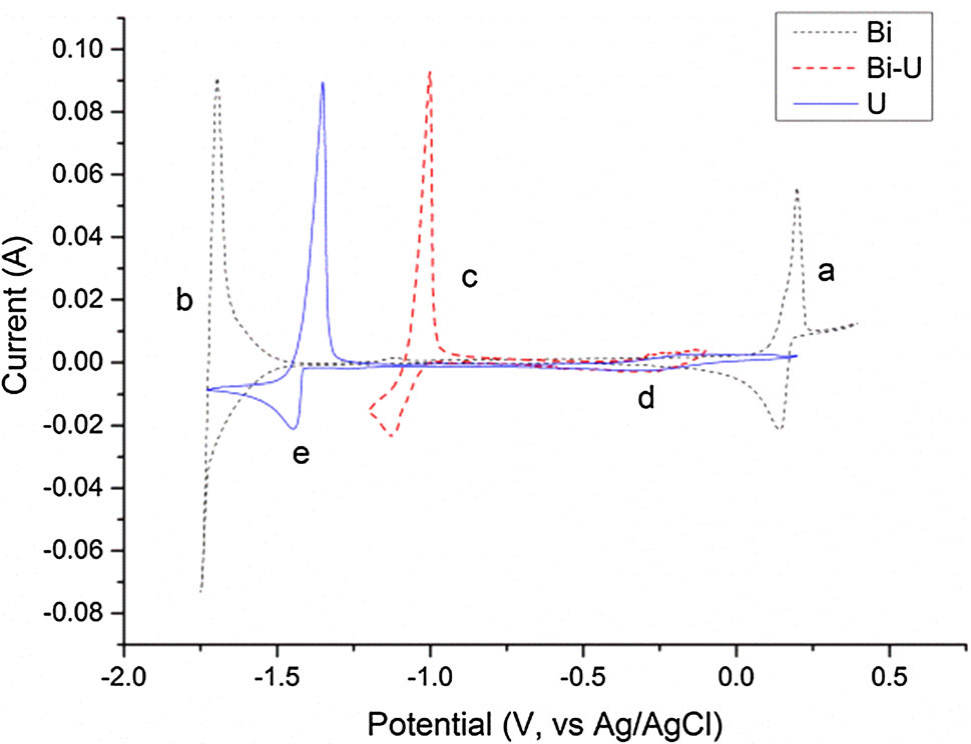
Cyclic voltammograms of BiCl_3_ dissolved in LKE (black dashed line; Assigned redox processes—a: Bi/Bi^3+^, b: Bi_x_Li_y_/Bi^3+^), partway through the reaction of BiCl_3_ with excess U metal in LKE (red dashed line; Assigned redox process—c: Bi_x_U_y_/U^3+^) and at completion of the reaction of BiCl_3_ with U metal in LKE (blue line; Assigned redox processes—d: U^3+^/U^4+^, e: U/U^3+^) {T = 450 °C; Scan rate = 0.1 V s^−1^; Working electrode: W rod; Counter electrode: W rod; Reference electrode: Ag/AgCl (1.5 wt% in LKE)}. (Color figure online)

Several minutes after the addition of BiCl_3_ to the melt, a deposition/stripping peak at 1.0 V (vs Ag/AgCl), which is typical for the formation of U–Bi alloys [34], was observed in the cyclic voltammogram shown in Fig. 10 (red curve). After bubbling argon through the melt, the OCP stabilized around − 0.6 V (vs Ag/AgCl), and the peaks in the CV at − 1.0 and 0.15 V (vs Ag/AgCl) were no longer observed while a new deposition/stripping peak appeared at − 1.6 V (vs Ag/AgCl) typical of the U/U^3+^ couple. A soluble–soluble wave of relatively weak intensity was identified at − 0.4 V (vs Ag/AgCl) which is assigned to the U^4+^/U^3+^ couple [19, 34].

From these results, we can deduce that the Bi^3+^ in solution reacts with the uranium metal to initially form what appears to be U(IV). Since the value of the OCP of the solution is higher than the U^3+^/U^4+^ potential, it is thought that the uranium metal is directly oxidized to U(IV), with no evidence for the formation of any significant quantities of U(III). Once the melt was bubbled with argon, the redox couples for Bi/Bi^3+^ and uranium bismuth alloys were not observed on the CVs of the melt, indicating Bi(III) was no longer present. Meanwhile characteristic peaks for the presence of uranium in the salt were observed. The measured OCP is concordant with W in contact with UCl_3_ in LiCl–KCl eutectic.

### Redox mechanism

The results described present a clear picture of the different reactions taking place in the melt. Firstly, the BiCl_3_ dissolves in the LiCl–KCl eutectic, as shown by the CV measurement in Fig. 8 and the OCP jump in Fig. 9, which has been identified by Volkov et al. by absorption spectroscopy as BiCl_4_^−^ [28]. Initially, the dissolved Bi(III) reacts with the uranium metal to form U(IV) in the bulk melt as explicated in Eq. (5) and observed by OCP measurement and absorption spectroscopy (Figs. 4 and 5). Once the concentration of U(IV) in the melt is high enough it combines with the uranium metal to form U(III), as shown by the increase of the U(III) peak at 899 nm shown in Fig. 6. The U(III) is then oxidized to U(IV) by the Bi(III) in the bulk solution. This is in accordance with Eqs. (6) and (7) and is observed in the “smog chimney” localized presence displayed in Fig. 3.

5 $$ {\text{U}}_{\text{metal}} + \frac{4}{3}{\text{Bi}}^{3 + }_{\text{sol}} \to {\text{U}}^{4 + }_{\text{sol}} + \frac{4}{3}{\text{Bi}}_{\text{metal}} $$

6 $$ {\text{U}}_{\text{metal}} + 3{\text{U}}^{4 + }_{\text{sol}} \to 4{\text{U}}^{3 + }_{\text{sol}} $$

7 $$ {\text{U}}^{3 + }_{\text{sol}} + \frac{1}{3}{\text{Bi}}^{3 + }_{\text{sol}} \to {\text{U}}^{4 + }_{\text{sol}} + \frac{1}{3}{\text{Bi}}_{\text{metal}} $$

The proposed chemical processes for the oxidation of uranium metal in LiCl–KCl eutectic by BiCl_3_ enables to differentiate the possible outcomes in the final melt composition as a function of the BiCl_3_/U metal ratio and the necessity for thorough mixing of the melt to ensure the oxidation state purity of U(III) in the melt. Indeed the formation of U(IV) as an intermediary product, calls for the presence of excess uranium metal to ensure pure U(III) in the salt solution. The possibility to obtain a mixture of U(III) and U(IV) if the conditions are not well controlled should also be considered. This can easily be verified by measuring the OCP of the solution.

## Conclusion

The mechanism of the reaction of uranium metal with bismuth trichloride in LiCl–KCl eutectic has been studied. Experimental work was conducted and monitored by in-situ electrochemical measurements, absorption spectroscopy and visual observation. It is thought that the mechanism can be described by the reactions explicated in Eq. (5–7).

It was not possible to obtain conclusive spectroscopic information at the surface of the uranium metal, so it is not possible to totally rule out the formation of U(III) as a short lived intermediate from the reaction described in Eq. (5). The formation of U(IV) as an intermediate product will prevent the use of this method for industrial purposes as has been seen for the use of CuCl_2_ as oxidant because of its reactivity with the metallic vessel. On the other hand, the high vapour pressure of BiCl_3_ and the low toxicity of the by-product makes this route a very convenient way to produce dissolved, redox-pure U(III) in LiCl–KCl eutectic that is ideal for laboratory research purposes, as long as stoichiometry is controlled (i.e. 1:1 U_metal_:Bi^3+^ molar ratio) and the reaction melt is sufficiently mixed. Further work will study the kinetics of the different reactions identified and explore the effect of possible alloying reactions at the surface of the uranium metal on the reaction mechanism and the quality of the final reaction products.
